# Effectiveness of a multicomponent exercise training program for the management of delirium in hospitalized older adults using near-infrared spectroscopy as a biomarker of brain perfusion: Study protocol for a randomized controlled trial

**DOI:** 10.3389/fnagi.2022.1013631

**Published:** 2022-12-15

**Authors:** Lucía Lozano-Vicario, Fabiola Zambom-Ferraresi, Fabricio Zambom-Ferraresi, Antón de la Casa-Marín, Iranzu Ollo-Martínez, Mikel L. Sáez de Asteasu, Bernardo Abel Cedeño-Veloz, Joaquín Fernández-Irigoyen, Enrique Santamaría, Román Romero-Ortuno, Mikel Izquierdo, Nicolás Martínez-Velilla

**Affiliations:** ^1^Department of Geriatric Medicine, Hospital Universitario de Navarra (HUN), Pamplona, Spain; ^2^Navarrabiomed, Hospital Universitario de Navarra (HUN), Instituto de Investigación Sanitaria de Navarra (IdisNa), Universidad Pública de Navarra (UPNA), Pamplona, Spain; ^3^Clinical Neuroproteomics Unit, Navarrabiomed, Hospital Universitario de Navarra (HUN), Instituto de Investigación Sanitaria de Navarra (IdisNa), Universidad Pública de Navarra (UPNA), Pamplona, Spain; ^4^Global Brain Health Institute, Trinity College Dublin, Dublin, Ireland

**Keywords:** delirium, near-infrared spectroscopy, multicomponent intervention, older adults, physical exercise, geriatric acute care unit

## Abstract

Delirium is an important cause of morbidity and mortality in older adults admitted to hospital. Multicomponent interventions targeting delirium risk factors, including physical exercise and mobilization, have been shown to reduce delirium incidence by 30–40% in acute care settings. However, little is known about its role in the evolution of delirium, once established. This study is a randomized clinical trial conducted in the Acute Geriatric Unit of Hospital Universitario de Navarra (Pamplona, Spain). Hospitalized patients with delirium who meet the inclusion criteria will be randomly assigned to the intervention or the control group. The intervention will consist of a multicomponent exercise training program, which will be composed of supervised progressive resistance and strength exercise over 3 consecutive days. Functional Near-Infrared Spectroscopy (NIRS) will be used for assessing cerebral and muscle tissue blood flow. The objective is to assess the effectiveness of this intervention in modifying the following primary outcomes: duration and severity of delirium and functional status. This study will contribute to determine the effectiveness of physical exercise in the management of delirium. It will be the first study to evaluate the impact of a multicomponent intervention based on physical exercise in the evolution of delirium.

**Clinical trial registration:**

ClinicalTrials.gov. identifier: NCT05442892 (date of registration June 26, 2022).

## Introduction

Delirium is an acute onset and fluctuating syndrome that is characterized by disturbances in level of awareness, attention, and other cognitive functions (Helfand et al., 2021). It is highly prevalent among older adults across healthcare settings (8–17% reported in emergency departments, 20–29% in geriatric acute care, 13–50% in surgical patients, and 19–82% in intensive care units; Inouye et al., 2014). Furthermore, delirium is particularly important in older patients not only for its high incidence and prevalence but also for the great impact of its consequences including longer hospital stay, increased risk of institutionalization at discharge, higher cognitive and functional impairment, higher mortality, and greater healthcare costs (Jackson et al., 2017). In addition, geriatric inpatients often experience accelerated functional decline during hospitalization associated with long bed-rest episodes. Some studies have shown that more than 83% of these patients are bedridden and only 4% are permitted to stand or walk, which increases the risk of delirium and hinders its resolution once it is established (Covinsky et al., 2003; Brummel et al., 2014; Zisberg et al., 2015; Martínez-Velilla et al., 2016). Multicomponent interventions such as the Hospital Elder Life Program (HELP) have been shown to reduce delirium incidence in the acute care setting by 43%, by acting on modifiable risk factors such as dehydration, pain, sensory impairment, malnutrition, and immobility, compared to usual care (Hshieh et al., 2018; Wang et al., 2020; Burton et al., 2021).

However, there is little evidence on treatments for delirium. According to current clinical guidelines, the non-pharmacological approach focused on correcting the underlying causes should always be the first option, reserving pharmacological treatment for cases of extreme agitation [Davis et al., 2019; National Institute for Health and Care Excellence (NICE), 2022]. The scarce evidence is due to the fact that the pathophysiology of delirium remains unclear. Several mechanisms have been proposed to explain the development of delirium involving certain processes such as neuroinflammation, neuronal damage, neurotransmitter disturbance, and acute cerebral failure caused by hypoxia-ischemia. In the hypoxia-ischemia theory, there is a vascular dysfunction that produces endothelial injury and blood–brain barrier (BBB) damage, causing low oxygen delivery to the brain parenchyma and contributing to a metabolic insufficiency that allows delirium development (Wilson et al., 2020).

Physical exercise has been shown to improve cerebral blood flow, increasing neurogenesis and neuroplasticity through the release of neurotransmitters and neurotrophic factors such as insulin-like growth factor-1 (IGF-1) and Brain-Derived Neurotrophic Factor (BDNF; Ben-Zeev, 2022), providing synaptic transmission and improving cognitive function. Physical exercise also decreases the accumulation of amyloid plaques and tau protein which has been shown to improve cognitive functions such as attention, memory, executive tasks, and information processing speed (Erickson et al., 2019; Bliss et al., 2021; Cruz et al., 2022; Rai and Demontis, 2022; Silva et al., 2022). In fact, an individualized, multicomponent exercise training program may be an effective therapy for improving cognitive function in very old patients during acute hospitalization (de Asteasu et al., 2019, 2022), cognitive impairment (Venegas-Sanabria et al., 2022), and depression (Li et al., 2022). Therefore, we hypothesized that strategies that help improve oxygen supply to the brain, such as physical exercise, could be useful in treating delirium.

Although there are several techniques to monitor brain activity and cerebral blood flow (e.g., brain magnetic resonance, positron emission tomography, and electroencephalogram), Near-Infrared Spectroscopy (NIRS) could potentially be effective.

NIRS is a non-invasive physiological monitoring method that measures light absorbance to calculate oxy-hemoglobin (oxy-HB) and deoxy-hemoglobin (deoxy-HB), which provide an indirect measure of tissue oxygenation, often in the frontal cortex of the brain and muscle.

Some of the advantages that NIRS can offer are that is a non-invasive technology, which provides real-time continuous measurement of regional cerebral blood oxygenation and indirect blood flow indicating perfusion adequacy. Being a portable device, it can be moved to the place where the patient is, which facilitates its use. In addition, NIRS does not emit ionizing radiation and is less expensive than brain neuroimaging tests such as magnetic resonance imaging, providing added advantages such as the analysis of functional brain parameters while the patient performs different physical or cognitive tasks (Sakudo, 2016; Nguyen et al., 2019; Milej et al., 2020; Hogue et al., 2021).

Even though NIRS is used in different areas of Medicine, it has reached its greatest interest in Intensive Care and Anesthesiology for monitoring brain perfusion to avoid hypoxemia, which is associated with cognitive dysfunction and delirium (Schoen et al., 2011; Hori et al., 2014). Some studies have recently been published using NIRS as a predictive biomarker of postoperative delirium with promising results, where it has been observed that patients with low levels of cerebral oxygen saturation prior to surgery and during surgical intervention, had a higher incidence of postoperative delirium (Wood et al., 2017; Khan et al., 2021; Semrau et al., 2021; Susano et al., 2021). On the other hand, NIRS has been used to monitor hemodynamic response in the prefrontal cortex region and in lower-limb muscle tissue doing physical exercise and functional tasks in acutely hospitalized older patients (de Asteasu et al., 2021).

In spite of the growing number of studies published on the application of NIRS in delirium over the last few years, there are currently significant limitations so this evidence should be interpreted with caution (Chen et al., 2020). These studies often include a low sample size, are usually carried out in the field of surgery or the Intensive Care Unit (ICU), are heterogeneous in their composition, and have a high risk of bias. In addition, most of them do not consider the peculiarities of the older population, which is where delirium is most frequent. In most studies, essential information about older adults is not systematically collected, including geriatric syndromes (frailty, falls, and malnutrition), predisposing factors (comorbidity, functional, and cognitive status), and precipitating factors of delirium (pain, polypharmacy, use of urinary catheters).

The objective of this clinical trial is to evaluate the effect of a multicomponent exercise program on the development of delirium assessing cerebral and muscle perfusion using NIRS in hospitalized older adults. This study may help to understand the mechanisms underlying delirium, which are not yet totally clear in the literature, considering tissue oxygenation hypothesis.

## Materials and methods

### Study design

**Figure 1:**
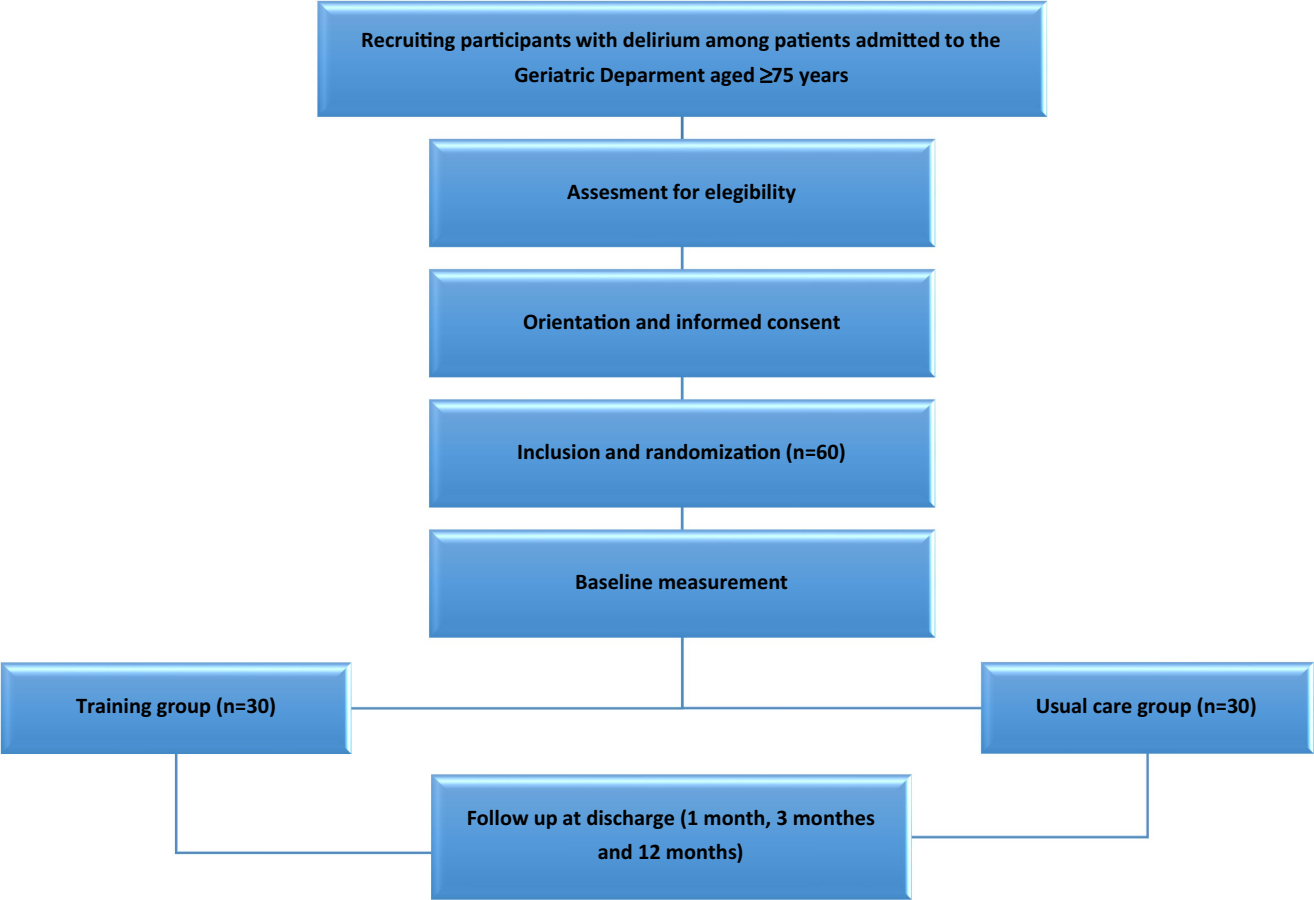
Flow diagram of the study protocol.

This study is a randomized clinical trial conducted in the 40-bedded Acute Geriatric Unit (AGU) of Hospital Universitario de Navarra (Pamplona, Spain). Hospitalized patients who meet the inclusion criteria will be randomly assigned to the intervention or control group. The study flow diagram is shown in Figure 1. After obtention of written informed consent, patients will be randomly assigned to either the intervention or control group. The data for both the intervention group and the control group will be obtained at five different times: the initial visit during the acute hospitalization, at discharge and at 1, 3, and 12 months after discharge through phone call and clinical history. The times of measurement of the different outcomes is shown in Table 1.

**Table 1:** Time of measurement of the different variables on the subjects of the study.

Measurement	T1 baseline	T2 daily	T3 After training or control period	T4 1 month	T5 3months	T6 12 months
Barthel Index	X		X	X	X	X
Lawton and Brody Scale	X			X	X	X
Hierarchical Assessment of Balance and Mobility (HABAM)	X	X	X			
Handgrip strength Short Physical Performance Battery (SPPB)	X X		X X			
1RM (leg press, chest press and knee extension)	X		X			
Muscle power 10 repetitions at 50% 1RM in leg press	X		X			
Trail making test-part A (TMT-A)	X		X			
Regional cerebral oxygen saturation (Scto2) in the forehead and vastus lateralis at rest, TMT-A, 1RM, and 10 reps x 50% 1RM	X		X			
Delirium assessment (4AT)	X	X	X			
Delirium severity (MDAS)	X	X	X			
Delirium Motor Subtype Scale (DMSS)	X	X	X			
Global Deterioration Scale (GDS/FAST)	X			X	X	X
Informant questionnaire on cognitive decline in the elderly (*IQCODE*)	X			X	X	X
Yesavage Geriatric Depression Scale	X					
Geriatrics syndromes	X	X	X	X	X	X
FRAIL scale and Clinical Frailty Scale (CFS)	X			X	X	X
Mini nutritional assessment (MNA)	X					
Falls	X	X	X	X	X	X
Quality of life (EQ-5D)	X			X	X	X
Pharmacological treatment and drug burden index (DBI)	X		X	X	X	X
Laboratory parameters	X		X			

Peripheral blood (PB) samples will be obtained from all patients at baseline and at discharge. EDTA blood collection tubes (Vacuette®, Greiner Bio-One) will be used. All PB samples will be centrifuged in a fixed-angle rotor at 3,300 *rpm* for 10 min at room temperature. After centrifugation, the serum in the upper layer will be carefully extracted from the plasma in the bottom layer, divided into 100 μL, and immediately stored at −80°C. Plasma and buffy coat will be also extracted and stored in polypropylene plastic tubes at −80°C.

We aim to examine the brain function during delirium and the effects of an intra-hospital exercise program on the prefrontal cortex region and on muscle function with the use of NIRS. Regional oxygen saturation (rSO2) in the forehead and vastus lateralis muscle will be recorded using NIRS using the NIRO-200NX C10448 monitor (Hamamatsu, Japan) by placing one optode on the patient’s forehead above the eyebrow and the other optode on the vastus lateralis muscle (Koyama et al., 2013; Udina et al., 2020). The measurements will be made with the patients resting in the sitting position during 60 s, doing trail making test part A and the assessment of 10 reps x 50% 1RM at the beginning of the study and the 4th day of the study, in both intervention and control groups. There are some factors which may alter NIRS parameters because they influence cerebral perfusion such as drugs, blood pressure, hemoglobin, or oxygen saturation. Although we will evaluate all of them, this is an important limitation of the study.

Delirium will be assessed using the European Spanish version of the 4AT (Delgado-Parada et al., 2022) daily during hospitalization until discharge. This tool has been validated for the Spanish population and is a reliable instrument for delirium detection in older patients. The 4AT scale is designed to be used as a delirium detection tool in general clinical settings. The 4AT has four parameters: Alertness, Abbreviated mental test-4 (AMT4), Attention (months backward test), and Acute change or fluctuating course. The score range is 0–12, with scores of 4 or more suggesting possible delirium. Scores of 1–3 suggest possible cognitive impairment (Jeong et al., 2020; Tieges et al., 2021). Delirium severity will be evaluated daily with the Memorial Delirium Assessment Scale (MDAS) which is also validated in Spain (Noguera et al., 2014; Barahona et al., 2018). MDAS was designed to diagnose delirium as well as classify delirium severity. The instrument reflects delirium diagnostic criteria from DSM. It has 10 severity items rated 0 to 3 points for a maximum total score of 30 points, with higher scores representing more severe delirium (Jones et al. 2019).

The protocol employs relevant standard protocol items for clinical trials according to the SPIRIT 2013 statement (Chan et al., 2013) and follows the CONSORT statement (Moher et al., 2001). The trial is registered at ClinicalTrials.gov, identifier NCT05442892. This study was approved by the Navarra Clinical Research Ethics Committee (PI_2021/94).

### Study participants and eligibility criteria

Medical inpatients admitted to the AGU of Hospital Universitario de Navarra (Pamplona, Spain) between February 2022 and February 2023.

The inclusion criteria are:

– Age: 75 years or older with delirium during hospitalization.
– Able to ambulate with or without personal/technical assistance.
– Barthel Index ≥45 points 2 weeks before admission.
– Informed consent by patients (if possible), relatives, or legal representatives.

The exclusion criteria are:

– Duration of hospitalization <5 days.
– Severe dementia (GDS 6–7).
– Terminal illness (life expectancy less than 3 months).
– Any factor precluding performance of physical exercise. These factors include:
– Acute myocardial infarction in the past 3 months or unstable angina
– Severe heart valve insufficiency.
– Arrhythmia or uncontrolled arterial hypertension.
– Pulmonary embolism in the past 3 months.
– Hemodynamic instability.
– Pathology that could interfere with NIRS registration:
– Facial dermal pathology (front).
– Acute intracranial pathology (hemorrhages, cerebral infarcts).

### Randomization and blinding

The study participants will be randomized^1^ into intervention or control group. Assessment staff will be blinded to the participant randomization assignment, as well as to the main study design and to what changes we expect to occur in the study outcomes in each group. It will not be possible to conceal the group assignment from the staff involved in the training of the intervention group. Patients and their families will be informed of the random inclusion in one group but will not be informed as to which group they belong.

### Sample size

Assuming a standard deviation in MDAS scale of 6 points, for a power of 80% and a significance level of 0.05, 24 patients per group (a total of 48) will be necessary to detect a mean difference of 5 points between groups. Assuming 20% losses, 30 patients per group will be necessary.

### Statistics

The baseline value of the included variables will be described for the whole sample and separated by group using frequencies and percentages for the categorical ones and *via* mean and standard deviation or median and interquartile range for continuous ones. In order to assess the extent of the therapeutic effect, we will compute for every patient the difference between final and initial levels of the continuous outcome variables. Then, these differences will be compared by intervention group, using *t*-tests or Mann–Whitney U tests. For the mortality outcome, the percentage mortality of both groups will be compared using the Chi-square test. In case of observing relevant differences at baseline, these comparisons will be adjusted by the baseline value, using linear models or generalized linear models. The level of statistical significance will be 0.05. Data will be analyzed with SPSS package 28.0.

### Detailed description

#### Usual care group (control)

Participants randomly assigned to the usual care group will receive normal hospital care, which includes physical rehabilitation when needed.

#### Intervention group (training)

**Figure 2:**
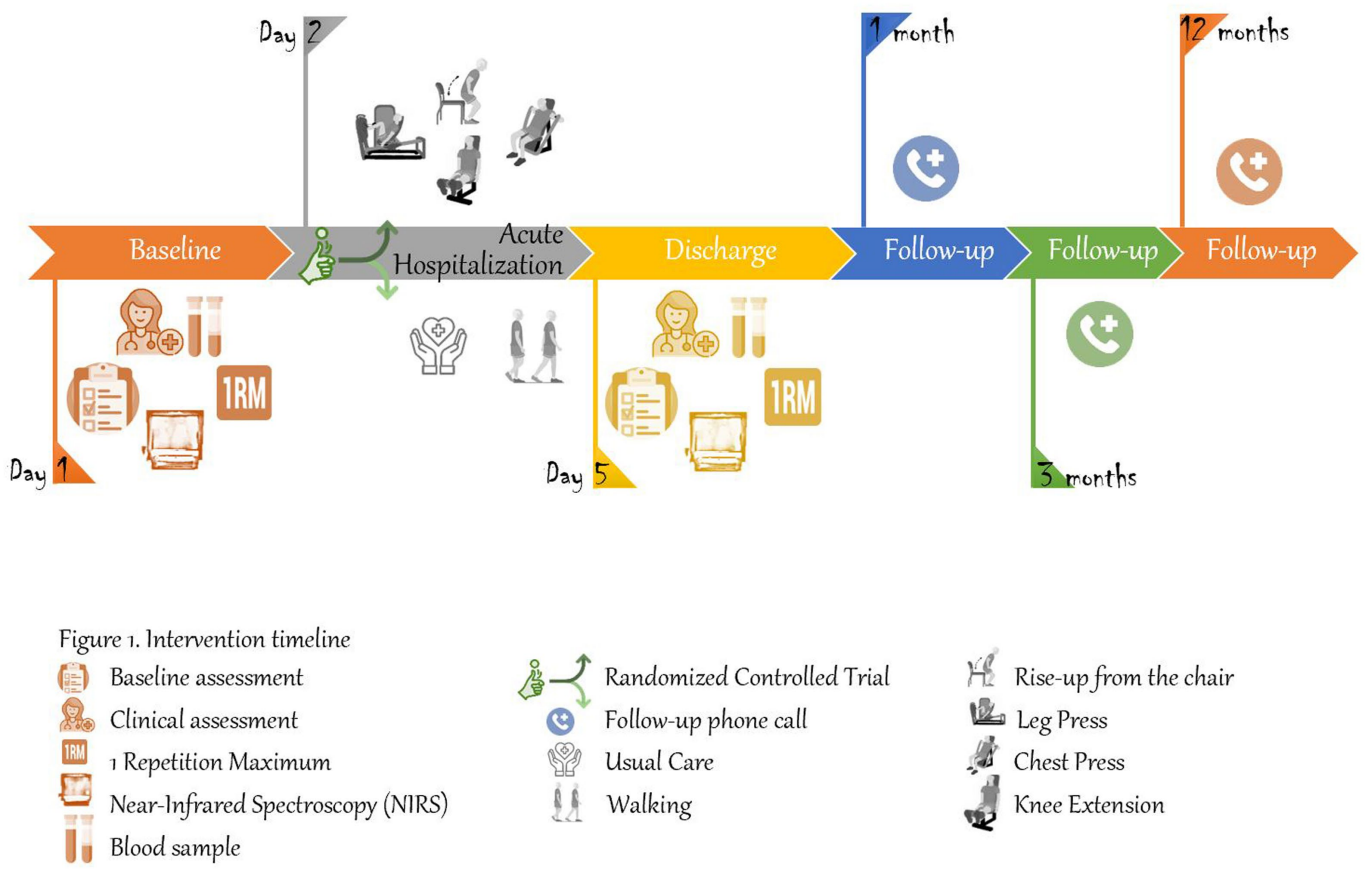
Intervention timeline.

The intervention will consist of a multicomponent exercise training program adapted from Vivifrail to prevent muscle weakness and falls (Izquierdo et al., 2017; Casas-Herrero et al., 2022) and the training protocol was detailed in previous literature (Martínez-Velilla et al., 2015, 2016, 2019, 2022). It will be composed of supervised progressive resistance exercise training, balance training, and walking for 3 consecutive days. During the training period, patients will be trained in 30 min sessions once a day (morning). The supervised multicomponent exercise training program will be comprised of upper and lower body strengthening exercises, tailored to the individual’s functional capacity, using weight machines and aiming for 2–3 sets of 8–10 repetitions at an intensity of 50–70% of 1RM (Matrix, Johnson Health Tech, Ibérica, S.L. Torrejón de Ardoz, Madrid, Spain). A “1RM” signifies the maximum resistance a person can move in one repetition of an exercise. The resistance exercises focused on the major upper and lower limb muscles. On the second and third training days, patients do 2 sets of 10 chair squats. Each resistance training session will include 2 exercises for the leg extensor muscles (bilateral leg press and bilateral knee extension machines) and 1 exercise for upper limbs (seated bench press machine). During the progressive resistance training, instruction will be provided to the participants to perform the exercises at a high velocity of motion. However, care will be taken to ensure that exercises are executed with correct form. In the first assessment, the patients will warm up with specific movements for the exercise test. Each subject’s maximal load will be determined in no more than five attempts. During all neuromuscular performance tests, a strong verbal encouragement will be given to each subject to motivate them to perform each test action as optimally and rapidly as possible. Three experienced physical trainers (FZF, IOM, and ADM) will carefully monitor and supervise all training sessions and provide instruction and encouragement. Adherence to the exercise intervention program will be documented in a daily register of sessions and adverse events including muscle pain, fatigue, falls and general aches will be recorded by the training staff. Participants and their family members will be carefully familiarized with the training procedures in advance. The intervention exercises are detailed in Table 2 and the training protocol is shown in Figure 2.

**Table 2:** Intervention group exercises.

Exercise	Day 1	Day 2	Day 3	Day 4
Rises from a chair	1 × 5	2 × 10	2 × 10	1 × 5
Leg press	1RM + 2 × 10 (50% 1RM)	3 × 10 (60% 1RM)	3 × 8 (70% 1RM)	1RM + 1 × 10 (50%1RM)
Chest press	1RM + 2 × 10 (50% 1RM)	3 × 10 (60% 1RM)	3 × 8 (70% 1RM)	1RM
Leg extension	1RM + 2 × 10 (50% 1RM)	3 × 10 (60% 1RM)	3 × 8 (70% 1RM)	1RM

### Outcome measures

#### Primary outcome

- Duration and severity of delirium during the hospitalization between both intervention and control group and the change in functional status: 4AT and MDAS scale.
- Functional capacity of patients will be evaluated by the Short Physical Performance Battery (SPPB), which evaluates, balance, gait ability, and leg strength using a single tool. The total score will range from 0 (worst) to 12 points (best). The SPPB test has been shown to be a valid instrument for screening frailty and predicting disability, institutionalization, and mortality (Guralnik et al., 1994). Daily functional status will be also assessed with the Hierarchical Assessment of Balance and Mobility (HABAM) with is an instrument that provides a clinical assessment of in-bed mobility, transfers, and ambulation (Gual et al., 2019; Richardson et al., 2021). The lowest number, a value of 0, is equal to the lowest or no performance. Changes in these abilities can then be compared with patient progress.
- Regional oxygen saturation (rSO2) will be measured using NIRS (Luo et al., 2018; Pfeifer et al., 2018).

#### Secondary outcome

- Cognitive status: Global Deterioration Scale (GDS) which describes 7 clinically distinguishable global stages, from normality to severe dementia of the Alzheimer type (Reisberg et al., 1982; Auer and Reisberg, 1997) and the Informant Questionnaire on Cognitive Decline in the Elderly short form (IQCODE) which is a 16-question form, each question is scored from 1 (much improved) to 5 (much worse) and a cutoff point (average score) of 3.31/3.38 achieves a balance of sensitivity and specificity of cognitive impairment (Blandfort et al., 2020; Harrison et al., 2021).
- Functional status: Barthel Index of independence during activities of daily living (ADLs). This index ranges from 0 worst to 100 best (Barthel, 1965).
- Mortality.
- Quality of life: EuroQol Scale-5D. This instrument measures 5 dimensions of health status: mobility, self-care, usual activities, pain/discomfort, and anxiety/depression (Herdman et al., 2001; García-Gordillo et al., 2015).
- Use of health sources: New admissions to the hospital, admission to nursing homes, visits to the general practitioner, and to the emergency department.
- Falls.

## Discussion

Given that the pathophysiology of delirium remains unclear and its pharmacological treatment once established has not been shown to be effective in addition to having serious side effects (extrapyramidal symptoms, sedation, arrhythmias…) physical exercise, due to its anti-inflammatory component and improvement of cerebral perfusion, can open as a new therapeutic option to explore. Other important aspect of our trial is the inclusion of older patients with mild cognitive decline and dementia. So far, most of trials in aged frail participants with these conditions are routinely excluded. The inclusion of participants with cognitive impairment in addition to frailty makes the trial novel with notable external validity compared with other previous trials in assessing the effect of individualized exercise programs on functional capacity, activities of daily living, and cognitive function. This study will both advance delirium-related knowledge and improve health outcomes through a program based on physical exercise. Moreover, this project will try to find new biomarkers of delirium that could be extrapolated to the usual clinical practice and help in its monitoring. Due to the high prevalence of delirium in this population and its serious consequences on morbidity and mortality, this intervention opens the possibility of a new therapeutic approach that can mitigate its impact. If our hypothesis is correct and shows that a multicomponent, individualized, and progressive exercise program in hospitalized older adults with delirium improves cognitive and functional status, a possible new targeted and therapeutic tool during hospitalization could be developed to implement delirium management.

## Trial status

The trial commenced recruitment on 7 February 2022 and is currently open for recruitment. Recruitment will cease when 60 participants have been randomized. It is anticipated this target will be reached by February 2023.

## Ethics statement

This study follows the principles of the Declaration of Helsinki (World Medical Association) and was approved by the Navarra Clinical Research Ethics Committee on September 15, 2021 (PI_2021/94). The patients/participants provided their written informed consent to participate in this study.

## Author contributions

L-LV, FabiZ-F, AC-M, and IO-M developed the protocol in consultation with FabrZ-F, MS, MI, and NM-V. LL-V, FabiZ-F, AC-M, and IO-M were involved in the recruitment and evaluation of the patients. All authors contributed to the article and approved the submitted version.

## Funding

NM-V received funding from “la Caixa” Foundation (ID 100010434), under agreement LCF/PR/PR15/51100006.

## Conflict of interest

The authors declare that the research was conducted in the absence of any commercial or financial relationships that could be construed as a potential conflict of interest.

## Publisher’s note

All claims expressed in this article are solely those of the authors and do not necessarily represent those of their affiliated organizations, or those of the publisher, the editors and the reviewers. Any product that may be evaluated in this article, or claim that may be made by its manufacturer, is not guaranteed or endorsed by the publisher.

## Supplementary material

The Supplementary material for this article can be found online at: https://www.frontiersin.org/articles/10.3389/fnagi.2022.1013631/full#supplementary-material

## Footnotes

^1^ www.randomizer.org
