# Supplementary material for: Effectiveness of a multicomponent exercise training program for the management of delirium in hospitalized older adults using near-infrared spectroscopy as a biomarker of brain perfusion: Study protocol for a randomized controlled trial
Source: Front Aging Neurosci. 2022 Dec 15;14:1013631. doi: 10.3389/fnagi.2022.1013631 (PMC9797855; doi:10.3389/fnagi.2022.1013631)
Supplement: Supplementary file 2 [file Data_Sheet_2.docx]

Supplementary Material 2

Items from de World Health Organization Trial Registration Data Set

| **Data category** | **Information** |
| --- | --- |
| Primary registry and trial identifying number | ClinicalTrials.gov NCT05442892 |
| Date of registration in primary registry | 26 June, 2022 |
| Secondary identifying numbers | -- |
| Source(s) of monetary or material support | “La Caixa” Foundation (ID 100010434) |
| Primary sponsor | “La Caixa” Foundation |
| Secondary sponsor(s) | -- |
| Contact for public queries | Lucía Lozano Vicario, MD, email: lucia.lozanovicario@gmail.com |
| Contact for scientific queries | Lucía Lozano Vicario, MD, Geriatric Unit, Hospital Universitario de Navarra, Pamplona (Spain) |
| Public title | Effectiveness of a multicomponent exercise training programme for the management of delirium in hospitalized older adults using Near-Infrared Spectroscopy (NIRS) as a biomarker of brain perfusion: study protocol for a randomized controlled trial |
| Scientific title | Exercise training in older adults with delirium -randomized controlled trial |
| Countries of recruitment | Spain |
| Health condition(s) or problem(s) studied | Delirium, frailty |
| Intervention(s) | *Control group*: normal hospital care  *Intervention group*: supervised and individualized multicomponent exercise training program |
| Key inclusion and exclusion criteria | The inclusion criteria are:   - Age: 75 years or older with delirium during hospitalization - Able to ambulate with or without personal/technical assistance - Barthel Index > 45 points two weeks before admission - Informed consent by patients (if possible), relatives or legal representatives   The exclusion criteria are:   - Duration of hospitalization < 5 days - Severe dementia (GDS 6-7) - Terminal illness (life expectancy less than 3 months) - Any factor precluding performance of physical exercise. These factors include:   - Acute myocardial infarction in the past three months or unstable angina   - Severe heart valve insufficiency   - Arrhythmia or uncontrolled arterial hypertension   - Pulmonary embolism in the past 3 months   - Hemodynamic instability - Pathology that could interfere with NIRS registration:   - Facial dermal pathology (front)   - Acute intracranial pathology (hemorrhages, cerebral infarcts) |
| Study type | -Interventional  -Allocation: randomized, Intervention model: parallel assignment; Masking: double blinded  -Primary purpose: treatment  -Phase III |
| Date of first enrolment | February, 2022 |
| Target sample size | 60 |
| Recruitment status | Recruiting |
| Primary outcome(s) | -Duration and severity of delirium (timeframe: 12 months)  -Functional status (timeframe:12 months)  -Regional oxygen saturation (rSO2), (timeframe: 12 months) |
| Key secondary outcomes | -Cognitive status (timeframe:12 months)  -Mortality (timeframe:12 months)  -Quality of life (timeframe:12 months)  -Use of health sources (timeframe:12 months)  -Falls (timeframe:12 months) |
