# Supplementary material for: Effectiveness of a multicomponent exercise training program for the management of delirium in hospitalized older adults using near-infrared spectroscopy as a biomarker of brain perfusion: Study protocol for a randomized controlled trial
Source: Front Aging Neurosci. 2022 Dec 15;14:1013631. doi: 10.3389/fnagi.2022.1013631 (PMC9797855; doi:10.3389/fnagi.2022.1013631)
Supplement: Supplementary file 3 [file Data_Sheet_3.docx]

**Supplementary Material 3: Informed Consent**

**“Multicomponent exercise training programme for the management of delirium in hospitalized older adults using Near-Infrared Spectroscopy (NIRS) as a biomarker of brain perfusion”**

**Informed Consent form for patients and caregivers**

This Informed Consent Form is for men and women who attend Hospital Universitario de Navarra (HUN) in Pamplona (Spain) or their caregivers, and who we are inviting to participate in research on delirium. The title of our research project is “Multicomponent exercise training programme for the management of delirium in hospitalized older adults using Near-Infrared Spectroscopy (NIRS) as a biomarker of brain perfusion”.

Principal Investigator: Lucía Lozano Vicario

Organization: Navarrabiomed, Fundación Miguel Servet

Sponsor: Fundación “La Caixa”

Version 2

This Informed Consent Form has two parts:

1. Information Sheet (to share information about the research with you)
2. Certificate of Consent (for signatures if you agree to take part)

You will be given a copy of the full Informed Consent Form

**PART I: INFORMATION SHEET**

**Introduction**

I am Lucía Lozano Vicario, a geriatrician working for Navarrabiomed Research Institute. We are doing research on delirium, which is a very common disease in this country. I am going to give you information and invite you to be part of this research. Before you decide, you can talk to anyone you feel comfortable with about the research.

There may be some words that you do not understand. Please ask me to stop as we go through the information and I will take time to explain. If you have questions later, you can ask them of me or the staff.

**Purpose of the research**

Delirium is an acutely disturbed state of mind characterized by restlessness, illusions, and incoherence. It is a common neuropsychiatric complication in hospitalized older adults which is associated with higher mortality and worse functional and cognitive recovery. Despite years of research, there are no effective alternatives for its treatment once established. The reason we are doing this research is to find out if a multicomponent programme based on physical exercise is better than usual care which is currently being used, during hospitalization.

**Type of Research Intervention**

This research will involve a questionnaire, two blood tests and a physical exercise programme during hospitalization.

**Participant selection**

We are inviting all older adults with delirium able to walk who attend Hospital Universitario de Navarra (HUN) to participate in this study.

**Voluntary Participation**

Your participation in this research is entirely voluntary. It is your choice whether to participate or not. Whether you choose to participate or not, all the services you receive at this hospital will continue and nothing will change. If you choose not to participate in this research project, you will be offered the treatment that is routinely offered in this hospital for delirium. You may change your mind later and stop participating even if you agreed earlier.

**Procedures and Protocol**

Because we do not know if doing physical exercise is better than usual care for the management of delirium during hospitalization among older adults, we need to compare both alternatives. To do this, we will put people taking part in this research into two groups. The groups are selected by chance, as if by tossing a coin.

Participants in one group will be trained with physical exercise while participants in the other group not (they will have usual care at Geriatric Unit which includes physical rehabilitation when needed). It is important that neither you nor we know which treatment you receive. This information will be in our files, but we will not look at these files until after the research is finished. This is the best way we have for testing without being influenced by what we think or hope might happen. We will then compare which of the two has the best results. The healthcare workers will be looking after you and the other participants very carefully during the study. If there is anything you are concerned about or that is bothering you about the research please talk to me or one of the other researchers.

*Description of the Process:* If you wish to participate, your medical history will be consulted and several questionnaires will be carried out in the first visit and 1, 3 and 12 months after discharge by telephone call. Likewise, a blood test will be taken on admission and prior to discharge along with your routine tests (at the end of the research, in 1 year, any left-over blood sample will be destroyed). Supervised and individualized physical exercise will be carried out during hospitalization (3 days, 30 minutes daily) by an expert physical therapist. This type of exercise is safe and we have a lot of experience in this field. Some participants in the research will not be trained instead, they will be given usual care which involves rehabilitation when needed. On the other hand, near-infrared spectroscopy (NIRS) will be used to measure your brain oxygenation at different times during hospital admission. This device is based on a non-invasive technology that does not cause any harm to the patient: it does not emit ionizing radiation and does not interfere with the use of other diagnostic, therapeutic or pharmacological techniques. Through some patches that are placed on the forehead, the cerebral blood flow is recorded.

**Duration**

The research takes place during hospitalization and 1 year after discharge in total. During that time, it will be necessary to do some questionnaires for follow-up by telephone call (1, 3 and 12 months after discharge).

**Side Effects**

Side effects during/after this exercise programme are very unusual muscle (pain, fatigue and general aches). However, we will follow you closely and keep track of any unwanted effects or any problems.

**Risks**

By participating in this research, it is possible that you will be at greater risk of having pain, fatigue or general aches. While the possibility of this happening is very low, you should still be aware of the possibility. We will try to decrease the chances of this event occurring, but if something unexpected happens, we will stop physical training and provide you whatever you need.

**Benefits**

If you participate in this research, you will benefit from the advantages of physical exercise: you will improve your functional status and strength. There may not be any benefit to older adults with delirium at this stage of the research, but future generations are likely to benefit.

**Reimbursements**

You will not be given any money or gifts to take part in this research.

**Confidentiality**

The information that we collect from this research project will be kept confidential. Information about you that will be collected during the research will be put away and no-one but the researchers will be able to see it. Any information about you will have a number on it instead of your name. Only the researchers will know what your number is and we will lock that information up with a lock and key.

**Sharing the Results**

The knowledge that we get from doing this research will be shared with you through community meetings before it is made widely available to the public. Confidential information will not be shared. There will be small meetings in the community and these will be announced. After these meetings, we will publish the results in order that other interested people may learn from our research.

**Right to Refuse or Withdraw**

You do not have to take part in this research if you do not wish to do so. You may also stop participating in the research at any time you choose. It is your choice and all of your rights will still be respected.

**Who to Contact**

If you have any questions you may ask them now or later, even after the study has started. If you wish to ask questions later, you may contact any of the following:

Lucía Lozano Vicario (email: [lucia.lozano.vicario@navarra.es](mailto:lucia.lozano.vicario@navarra.es))

This proposal has been reviewed and approved by Navarra Clinical Research Ethics Committee which is a committee whose task it is to make sure that research participants are protected from harm.

**PART II: CERTIFICATE OF CONSENT**

I have read the foregoing information, or it has been read to me. I have had the opportunity to ask questions about it and any questions that I have asked have been answered to my satisfaction. I consent voluntarily to participate as a participant in this research.

Name of Participant_____________________________________

Signature of Participant _________________________________

Date ___________________________

Day/month/year

I have witnessed the accurate reading of the consent form to the potential participant, and had the opportunity to ask questions. I confirm that the individual has given consent freely.

Name of witness_________________________

Signature of witness ______________________

Date ________________________

Day/month/year

The researcher/person taking consent:

I have accurately read out the information sheet to the potential participant. I confirm that the participant was given an opportunity to ask questions about the study, and all the questions asked by the participant have been answered correctly and to the best of my ability. I confirm that the individual has not been coerced into giving consent, and the consent has been given freely and voluntarily.

A copy of this ICF has been provided to the participant.

Name of Researcher/person taking the consent________________________

Signature of Researcher /person taking the consent__________________________

Date ___________________________

Day/month/year
